# Evaluating the Accuracy of Breast Cancer and Molecular Subtype Diagnosis by Ultrasound Image Deep Learning Model

**DOI:** 10.3389/fonc.2021.623506

**Published:** 2021-03-05

**Authors:** Xianyu Zhang, Hui Li, Chaoyun Wang, Wen Cheng, Yuntao Zhu, Dapeng Li, Hui Jing, Shu Li, Jiahui Hou, Jiaying Li, Yingpu Li, Yashuang Zhao, Hongwei Mo, Da Pang

**Affiliations:** ^1^Department of Breast Surgery, Harbin Medical University Cancer Hospital, Harbin, China; ^2^Harbin Engineering University Automation College, Harbin, China; ^3^Department of Ultrasound, Harbin Medical University Cancer Hospital, Harbin, China; ^4^Department of Epidemiology, Harbin Medical University, Harbin, China; ^5^Prenatal Diagnosis Center, The First Affiliated Hospital of Harbin Medical University, Harbin, China

**Keywords:** breast cancer, deep learning, ultrasound, cancer diagnosis, molecular subtype

## Abstract

**Background:** Breast ultrasound is the first choice for breast tumor diagnosis in China, but the Breast Imaging Reporting and Data System (BI-RADS) categorization routinely used in the clinic often leads to unnecessary biopsy. Radiologists have no ability to predict molecular subtypes with important pathological information that can guide clinical treatment.

**Materials and Methods:** This retrospective study collected breast ultrasound images from two hospitals and formed training, test and external test sets after strict selection, which included 2,822, 707, and 210 ultrasound images, respectively. An optimized deep learning model (DLM) was constructed with the training set, and the performance was verified in both the test set and the external test set. Diagnostic results were compared with the BI-RADS categorization determined by radiologists. We divided breast cancer into different molecular subtypes according to hormone receptor (HR) and human epidermal growth factor receptor 2 (HER2) expression. The ability to predict molecular subtypes using the DLM was confirmed in the test set.

**Results:** In the test set, with pathological results as the gold standard, the accuracy, sensitivity and specificity were 85.6, 98.7, and 63.1%, respectively, according to the BI-RADS categorization. The same set achieved an accuracy, sensitivity, and specificity of 89.7, 91.3, and 86.9%, respectively, when using the DLM. For the test set, the area under the curve (AUC) was 0.96. For the external test set, the AUC was 0.90. The diagnostic accuracy was 92.86% with the DLM in BI-RADS 4a patients. Approximately 70.76% of the cases were judged as benign tumors. Unnecessary biopsy was theoretically reduced by 67.86%. However, the false negative rate was 10.4%. A good prediction effect was shown for the molecular subtypes of breast cancer with the DLM. The AUC were 0.864, 0.811, and 0.837 for the triple-negative subtype, HER2 (+) subtype and HR (+) subtype predictions, respectively.

**Conclusion:** This study showed that the DLM was highly accurate in recognizing breast tumors from ultrasound images. Thus, the DLM can greatly reduce the incidence of unnecessary biopsy, especially for patients with BI-RADS 4a. In addition, the predictive ability of this model for molecular subtypes was satisfactory,which has specific clinical application value.

## Introduction

Breast cancer is the most common malignant tumor in women in China (1, 2). Breast ultrasound is more suitable for tumor discovery in Asian women considering the higher breast density (3, 4) and the younger age at diagnosis (5, 6). Patients with Breast Imaging Reporting and Data System (BI-RADS) 4a or higher findings are usually recommended to undergo core needle biopsy or surgery. BI-RADS has a wide range of possibilities to predict the presence of malignancies, but its false positive findings lead to unnecessary biopsies in a large number of individuals without breast cancer (7).

The combination of deep learning (8) and large datasets has shown good performance in the diagnosis of many diseases, including cancer (9–12). The deep learning model (DLM) takes the original image pixels and corresponding category labels in medical image data as inputs and does not require manual design features required by traditional methods but automatically learns features related to category classification (13).

Based on receptor status, breast cancers are divided into five subtypes (14). If the molecular subtype is identified before surgery, we can determine whether the patient is suitable for neoadjuvant treatment and which scheme should be more efficient. However, currently, we cannot obtain subtype information through traditional ultrasound examinations.

In addition to the differentiation of benign and malignant breast tumors from ultrasound images, previous studies have focused on the correlation between imaging features and molecular subtypes. Breast cancers with the triple-negative subtype were more likely to be associated with circumscribed margins and were less associated with calcifications (15–18). Human epidermal growth factor receptor 2 (HER2) (+) breast cancers usually show enhanced posterior acoustics on ultrasound images (15, 17). Tumors with posterior shadowing are often found in hormone receptor (HR) (+) HER2 (–) breast cancers (16, 19, 20). In addition, echogenic halos were frequently present in the HR (+) HER2 (–) subtype (17, 20). Due to the various imaging features of different subtypes, there is potential to predict molecular subtypes with DLM by analyzing only the ultrasound images.

In this study, we constructed a DLM based on ultrasound images. We obtained a higher accuracy for breast tumor diagnosis with the DLM than with radiologists. We obtained a good prediction for tumor molecular subtypes, which may provide more choices for therapy.

## Materials and Methods

This study was approved by the Institutional Review Board of Harbin Medical University Cancer Hospital. Because of its retrospective nature, the study was exempt from obtaining informed consent from patients.

### Datasets

**Figure 1:**
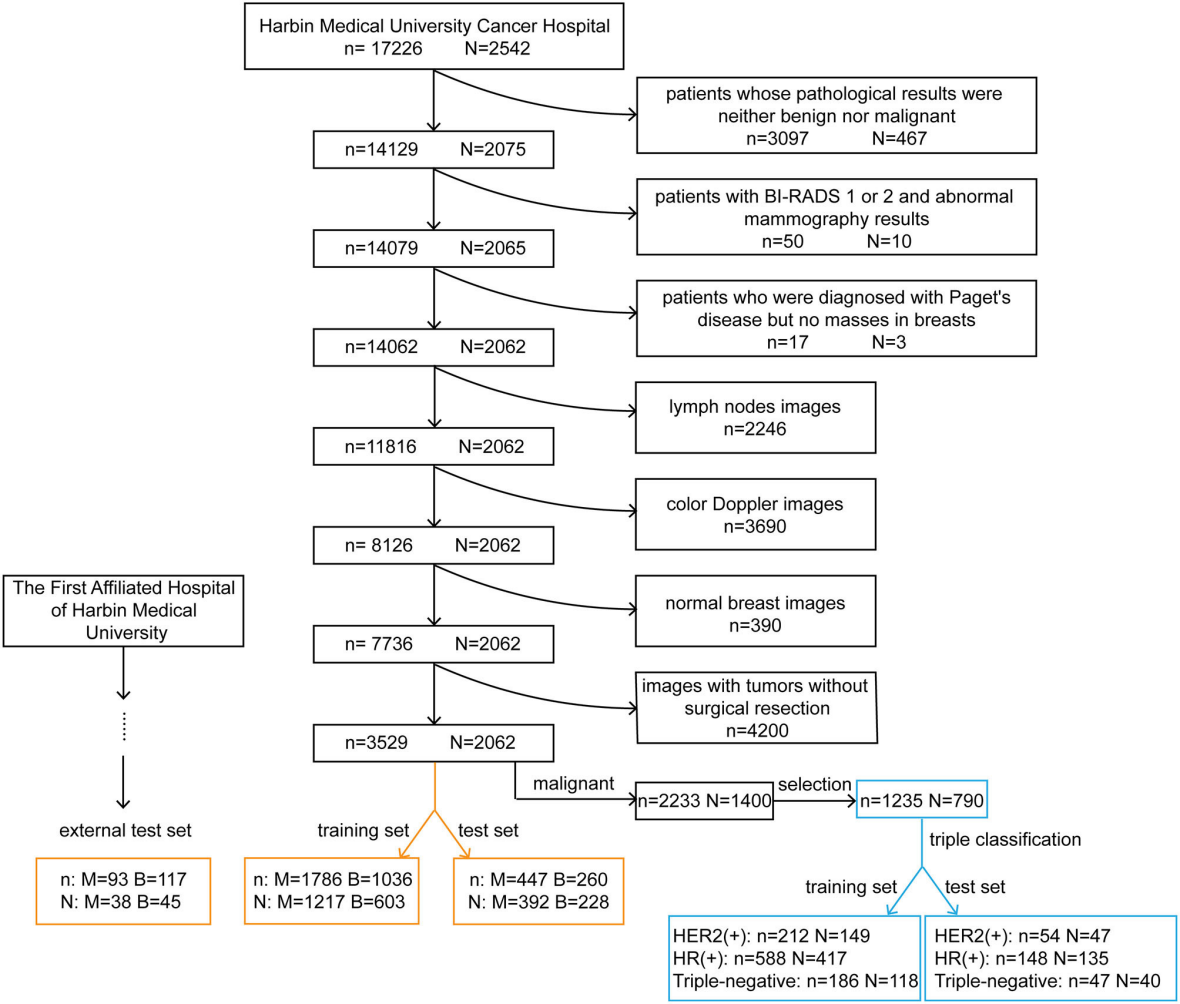
Cohort selection flowchart and the composition of the training, test and external test sets. The black rectangles represent the process of data selection. The orange rectangles represent the composition of datasets for the diagnosis of breast tumors. The blue rectangles represent the composition of datasets used to predict the molecular classification of breast cancer. *n*, the number of images; *N*, the number of patients; M, malignant; B, benign; HER2, human epidermal growth factor receptor 2; HR, hormone receptor.

We obtained original ultrasound images for the training and testing datasets from the breast image database of Harbin Medical University Cancer Hospital (a total of 17,226 images from 2,542 patients). All patients underwent surgical treatment with definitive pathological results. The cohort selection flowchart is shown in Figure 1. Patients in the external test set were enrolled from The First Affiliated Hospital of Harbin Medical University and were selected with the same criteria as those for the training and test sets. Exclusion criteria for the datasets are described in the Supplementary Material.

We selected ultrasound images of breast cancer patients with corresponding pathological results. We excluded tumors with incomplete immunohistochemistry results and then separated the tumors into molecular subtypes. Data on estrogen receptor (ER) and progesterone receptor (PR) expression were collected. Patients with positivity for either or both receptors were defined as being HR positive. According to the expression of two indicators, HR and HER2, we regrouped patients into three molecular subtypes: HER2 (+) subtype, HR (+) subtype and triple-negative subtype. HER2 (+) subtype = HR (+) HER2 (+) or HR (–) HER2 (+); HR (+) subtype = HR (+) HER2 (–); triple-negative subtype = HR (–) HER2 (–).

**Figure 2:**
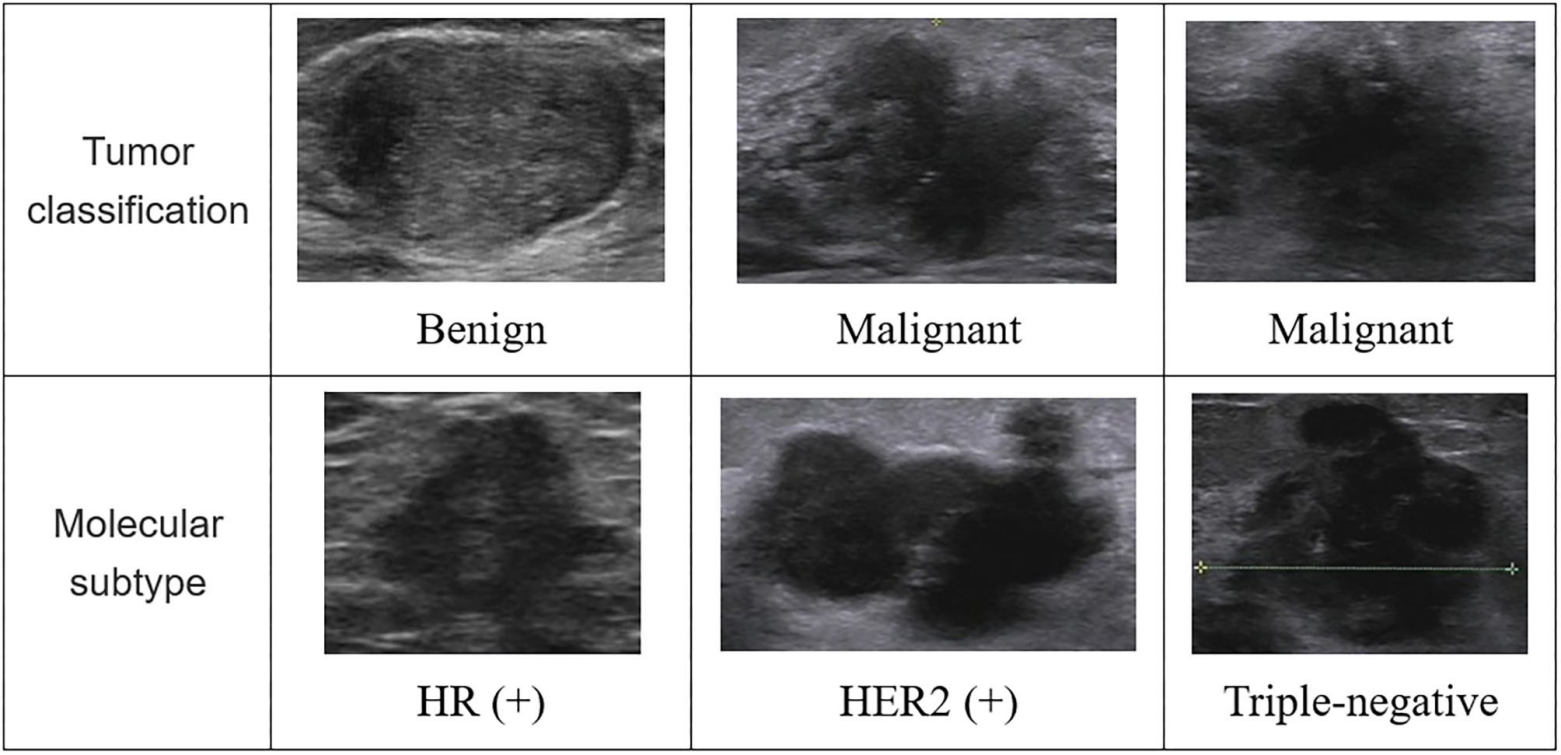
Some samples of breast ultrasound images from the datasets. In the first column, the samples were divided according to the diagnosis results of the tumor, which were benign and malignant; in the second column, the samples were classified according to molecular subtypes, which were divided into images of HR (+) subtype, HER2 (+) subtype, and triple-negative subtype. HR, hormone receptor; HER2, human epidermal growth factor receptor 2.

In Figure 2, we listed some samples of breast ultrasound images from the datasets, which were presented by classification of benign and malignant tumors and molecular subtypes.

The training and test sets were formed by a random sampling method at a ratio of 4:1. The compositions of these sets are shown in Figure 1. It is noteworthy that within the training set, we redivided it into a new training set and a validation set for model tuning and training at a ratio of 4:1. The optimal model obtained was tested with the test set and the external test set.

### Development of the DLM

All images in the datasets were 8-bit and 3-channel images, so they could be used as training set and test set images to fine-tune the deep convolutional neural network (DCNN) directly. Supplementary Figure 1 shows the whole process of breast ultrasound image analysis.

To obtain a better deep learning effect, the necessary image preprocessing algorithm was used to improve the image quality. In the image preprocessing of the Supplementary Material, we specifically described how to carry out preprocessing of breast ultrasound images.

Considering the amount of data compared with the depth of deep learning, we used data enhancement to enhance the diversity and generalization of the data. We used data enhancement in the Keras model, which could enhance the real-time data with the help of a central processing unit (CPU) during training. Due to the particularity of the ultrasound image data, we used four kinds of random operations to enhance the data: vertical rotation, horizontal rotation, center rotation, and scale reduction.

Because the data had enough high-quality samples after preprocessing and expansion, we used the deep learning Keras framework to transfer and fine-tune the Xception network, making it a network that could extract features for breast ultrasound images (21). The Xception convolution neural network (CNN) has trained more than 1.2 million images from the ImageNet large-scale vision recognition challenge (ILSVRC) knowledge base.

**Figure 3:**
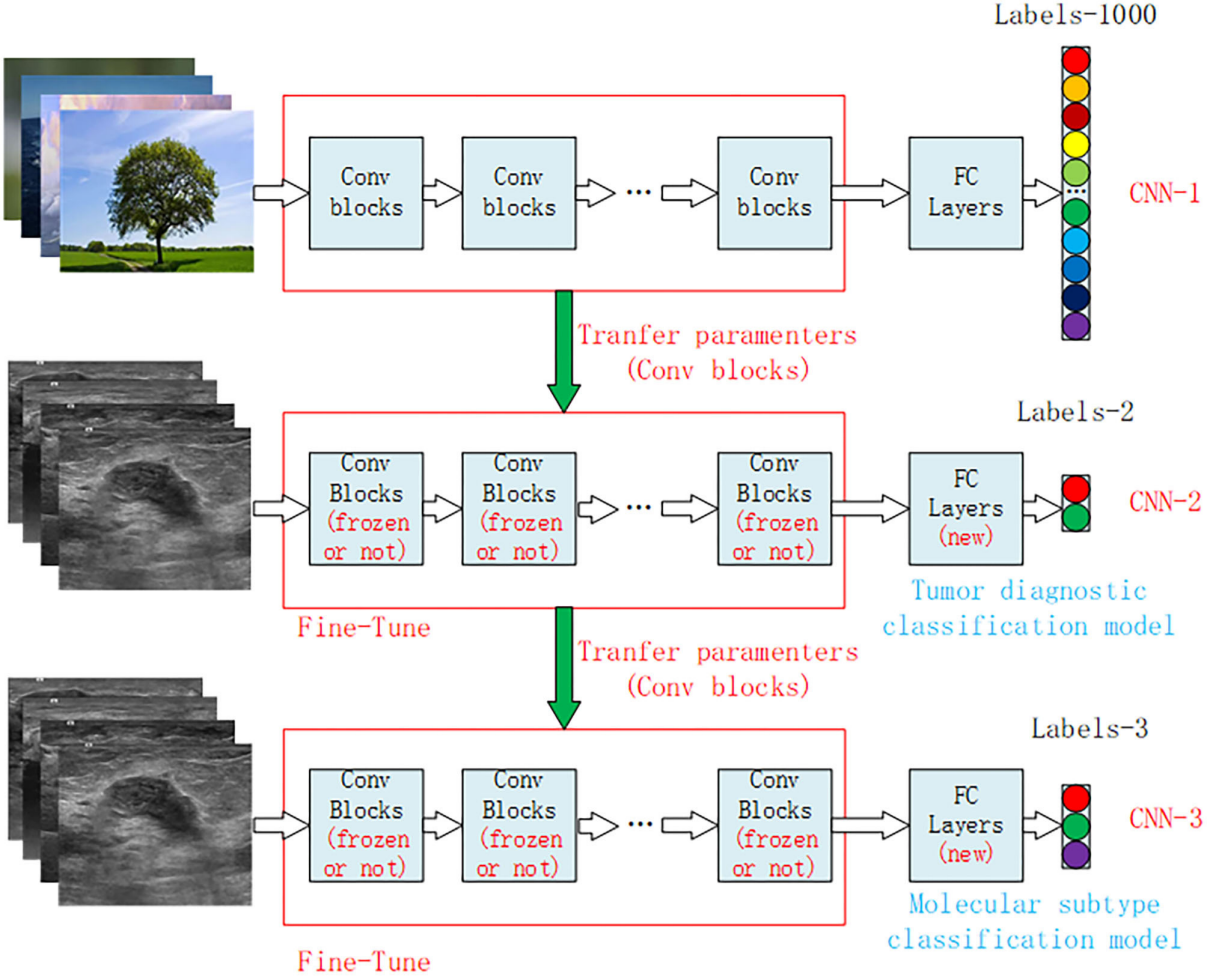
Structural diagram of the transfer learning model for breast cancer ultrasound images. CNN-1 represents the classification model trained on the ImageNet dataset, which is divided into 1,000 categories of natural images. CNN-2 is the benign and malignant tumor diagnostic model obtained by model transfer and retraining on the basis of the CNN-1 model, and CNN-3 is the classification model of tumor molecular subtypes obtained by model transfer and retraining on the basis of the CNN-2 model. CNN, convolution neural network.

The structure of the Xception model mainly consisted of a convolutional layer and a fully connected classification layer. Figure 3 shows the structure diagram of our model training, which mainly adopted the transfer learning method to train the model. CNN-1 represented the training model on the ImageNet dataset and outputted 1,000 classification results. The CNN-2 model was obtained through transfer learning of the CNN-1 model and used for the classification of benign and malignant breast tumors by ultrasound images. The specific model training method was to freeze the convolutional layer parameters of CNN-2 and then train the fully connected layer of the CNN-2 model. After the training was stable, we defrosted the convolutional layer for retraining to achieve the best effect. CNN-3 was a classification model of molecular subtypes, which was acquired from the transfer learning of the CNN-2 model. Two specific training parameters are shown in the Supplementary Material.

### Statistical Analysis

Evaluation of the DLM was performed with R version 3.5.1. For binary classification in discriminating breast cancer patients from controls, a classification matrix (caret version 6.0–80) and the receiver operating characteristic (ROC) curve (pROC version 1.13.0) were generated to visualize the diagnostic ability of the DLM. For the triple classification of the three molecular subtypes, a classification matrix and the ROC curve using a one-vs-all approach (multiROC version 1.1.1) were generated. The area under the curve (AUC), accuracy, sensitivity, and specificity were calculated to compare the predictive performance between the DLM and BI-RADS classification systems in the test and external test sets. *P* < 0.05 was considered to indicate a statistically significant difference. Moreover, the calculation of sample size is shown in the Supplementary Material.

## Results

### Cohort Composition

We divided the ultrasound images collected from Harbin Medical University Cancer Hospital into two sets (Figure 1). The training set was composed of 2,822 images, including 1,786 images from 1,217 patients with malignant tumors and 1,036 images from 603 patients with benign tumors. The test set was composed of 707 images, including 447 images from 392 patients with malignant tumors and 260 images from 228 patients with benign tumors. After applying the same exclusion criteria, external test set images were collected from The First Affiliated Hospital of Harbin Medical University, including 93 images from 38 patients with malignant tumors and 117 images from 45 patients with benign tumors.

Two datasets were used for molecular subtype prediction (Figure 1). The training set consisted of 212 images from 149 HER2 (+) subtype patients, 588 images from 417 HR (+) subtype patients and 186 images from 118 triple-negative subtype patients. The test set comprised 54 images from 47 HER2 (+) subtype patients, 148 images from 135 HR (+) subtype patients and 47 images from 40 triple-negative subtype patients.

### Performance in Diagnosis

**Figure 4:**
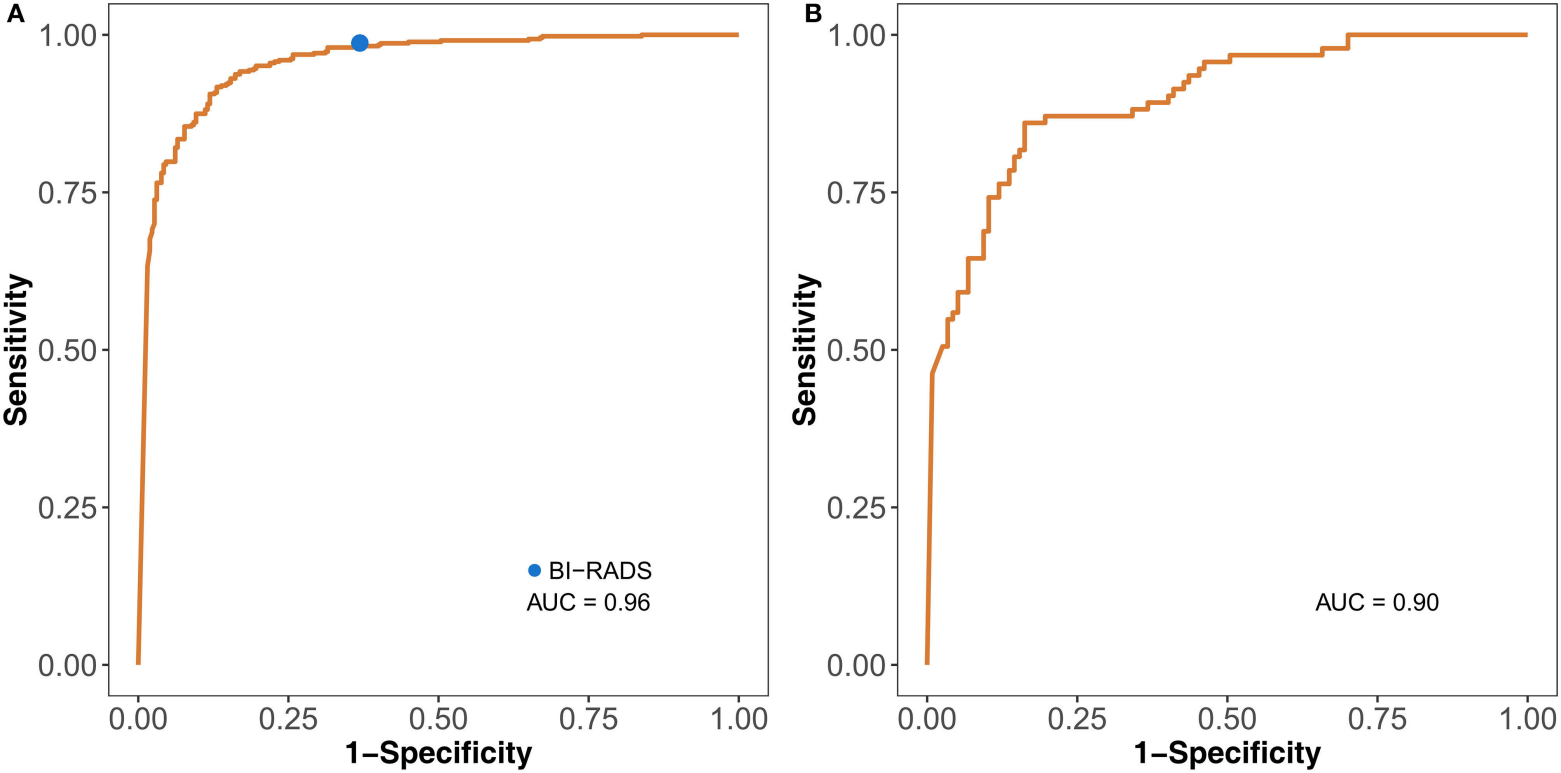
Identification performance of the DLM on the test set **(A)** and external test set **(B)**. The blue dot on the left ROC curve indicates the performance of BI-RADS. DLM, deep learning model; ROC, receiver operating characteristic; BI-RADS, Breast Imaging Reporting and Data System; AUC, area under the curve.

The DLM was more accurate (0.897, 95% CI: 0.872–0.918) than ultrasound doctors (0.856, 95% CI: 0.828–0.881) (*P* = 0.024) (Tables 1, 2). The doctors used the malignant probability and BI-RADS system to determine positive ultrasound findings. For sensitivities, 91.3 and 98.7% (*P* < 0.001) were achieved for the DLM and BI-RADS systems, respectively. However, the specificity of the DLM (86.9%) was significantly higher than that of BI-RADS (63.1%) (*P* < 0.001). This result indicates that the DLM may reduce the unnecessary biopsy of false positive findings with the BI-RADS system. For the test set, the AUC was 0.96. For the external test set, the AUC was 0.90 (Figure 4). These results suggest that the DLM has good performance in breast cancer diagnosis.

**Table 1:** Confusion matrices of the test set and external test set.

		**Pathology**			
		**Test set**		**External test set**	
		**+**	**−**	**+**	**−**
BI-RADS	+	441	96	–	–
	–	6	164	–	–
DLM	+	408	34	76	18
	–	39	226	17	99

*+, malignant; –, benign; BI-RADS, Breast Imaging Reporting and Data System; DLM, deep learning model*.

**Table 2:** Identification performance of BI-RADS and the DLM on the test set and external test set.

	**Test set**			**External test set**	
	**BI-RADS**	**DLM**	*P-*value^*^	**BI-RADS**	**DLM**
Accuracy	0.856	0.897	0.024	–	0.833
Sensitivity	0.987	0.913	<0.001	–	0.817
Specificity	0.631	0.869	<0.001	–	0.846
Positive predictive value	0.821	0.923	<0.001	–	0.809
Negative predictive value	0.965	0.853	<0.001	–	0.853
Kappa	0.666	0.779	–	–	0.663

*BI-RADS, Breast Imaging Reporting and Data System; DLM, deep learning model*;

* *chi-square test*.

### Reducing Unnecessary Biopsy

**Figure 5:**
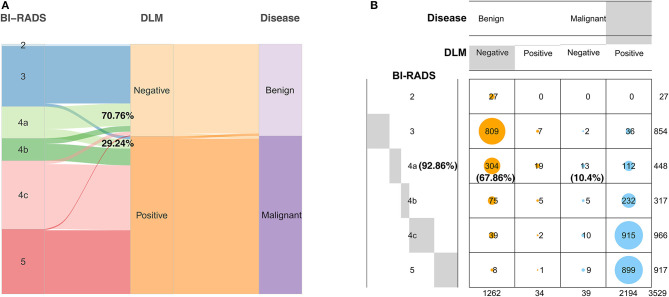
Diagnostic results of the DLM for breast tumors with different BI-RADS categorizations compared with the definitive pathological results. **(A)** The width of the extended branches corresponds to the size of the data; 70.76% represents the percentage of BI-RADS 4a patients diagnosed as benign by the DLM; 29.24% represents the percentage of BI-RADS 4a patients diagnosed as malignant by the DLM. **(B)** The numbers in this graph represent the number of images; 92.86% represents the diagnostic accuracy of BI-RADS 4a patients with the DLM; 67.86% represents the reduction rate of unnecessary biopsy in BI-RADS 4a patients with the DLM; 10.4% represents the false negative rate of BI-RADS 4a patients with the DLM. Negative means the diagnosis of the DLM is benign. Positive means the diagnosis of the DLM is malignant. DLM, deep learning model; BI-RADS, Breast Imaging Reporting and Data System.

Each ultrasound image had corresponding BI-RADS, DLM, and pathological results. The proportion of all patients with BI-RADS 4a judged as benign (70.76%) by the DLM was greater than that judged as malignant (29.24%) (Figure 5A). This result indicated that 70.76% of BI-RADS 4a patients did not need surgery when diagnosed using the DLM. The diagnostic accuracy for BI-RADS 4a patients reached 92.86%, and unnecessary biopsy was reduced by 67.86%, with a false negative rate of 10.4% for the DLM (Figure 5B). These findings suggest that the DLM can greatly reduce the incidence of unnecessary biopsy, especially for BI-RADS 4a patients with a low false negative rate.

### Molecular Subtype Prediction

**Figure 6:**
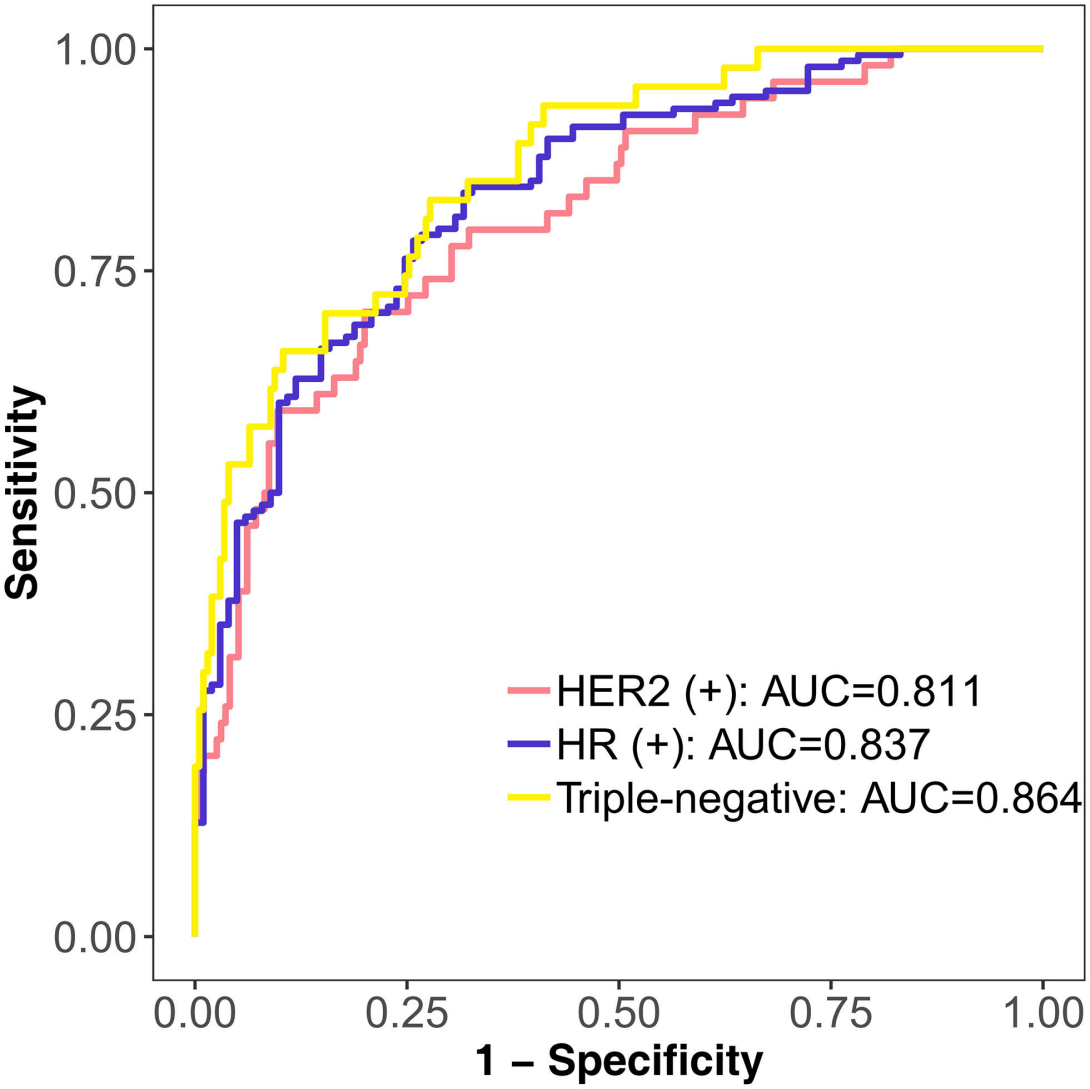
Performance of the DLM in identifying different molecular subtypes with triple classification on the test set. HER2 (+) subtype = HR (+) HER2 (+) or HR (–) HER2 (+); HR (+) subtype = HR (+) HER2 (–); triple-negative subtype = HR (–) HER2 (–). DLM, deep learning model; HER2, human epidermal growth factor receptor 2; HR, hormone receptor.

The DLM can be used not only in the diagnosis of breast cancer but also in the prediction of molecular subtypes. From the results of the triple classification, the triple-negative subtype reached the highest AUC of 0.864. The AUC of the HER2 (+) subtype was 0.811, and the AUC of the HR (+) subtype was 0.837 (Figure 6). Accuracy of the HR (+) subtype (85.14%) was significantly higher than that of the HER2 (+) subtype (50%) and triple-negative subtype (53.19%) (Supplementary Table 1).

## Discussion

We successfully established a DLM for breast cancer diagnosis and molecular subtype prediction based on ultrasound images. The accuracy of the DLM in the diagnosis of breast cancer was higher than that of BI-RADS, and the DLM performed well in both the test set and external test set. The DLM can apparently reduce unnecessary biopsy for patients with BI-RADS 4a. In the predictive results of the DLM for the molecular subtypes of breast cancer, we could see that its performance for various subtypes was ideal, and there were no significant disparities among them.

In many studies of cancer diagnosis, the accuracy of DLMs is higher than that of radiologists. Li et al. successfully developed a DCNN model for the diagnosis of thyroid cancer with ultrasound images. The accuracy was 89.8% with the DCNN model vs. 78.8% with radiologists (22). To classify invasive adenocarcinomas from preinvasive lesions, Wang et al. (23) developed a CNN model. The accuracy of the model (84%) was higher than that of three radiologists (radiologist 1: 80.2%; radiologist 2: 80.7%; and radiologist 3: 81.7%). He et al. used a CNN to predict the local recurrence of giant cell bone tumors. The accuracy of the CNN model was 75.0%, while the accuracy of radiologists was 64.3% (24). Our study had similar results. The DLM was more accurate (0.897, 95% CI: 0.872–0.918) than radiologists (0.856, 95% CI: 0.828–0.881) (*P* = 0.024). In summary, the DLM performed well and has the potential to provide better diagnostic results than radiologists.

At present, there are few studies on the diagnosis of breast cancer with DLMs based on ultrasound images. In recent studies, researchers used different DLMs to diagnose breast tumors on ultrasound images, and the one that performed best was selected after comparison. The purpose of these studies was to develop only a DLM for the classification of malignant and benign masses (25–29). In our study, we sought to develop a DLM not only for classifying masses but also for reducing unnecessary biopsy. Unnecessary biopsy was theoretically reduced by 67.86% with the DLM in BI-RADS 4a patients. Zhu et al. (30) developed a DLM based on breast MRIs that showed some predictive value for molecular subtypes. However, these researchers only considered the distinction between the luminal A subtype and all other subtypes. Unlike their model, our model can differentiate each molecular subtype and guide individualized treatment. To the best of our knowledge, this is the first study to apply a DLM to the prediction of molecular subtypes using ultrasound images.

The DLM is better than traditional methods in identifying benign and malignant breast tumors. It performs well with high AUC values and other indicators and reduces the burden of radiologists (31). DLMs do not require time-consuming tumor boundary labeling, which is a necessary step for traditional methods. In addition, the DLM can make better use of the hidden information around the tumor, which is ignored by traditional methods.

Currently, most studies use statistical analysis to obtain low-dimensional features of breast ultrasound images for molecular classification. These low-dimensional features are easily affected by the number and quality of the samples collected, making it difficult to mine and quantify the relationships between images and subtypes (32). However, deep learning methods can extract abstract features. All extracted features are high-dimensional features related to molecular classification. Although difficult to visualize, molecular classification is important and can greatly improve recognition accuracy.

There are limitations in our study. First, the training set data came from one hospital, and we did not summarize the basic information on patients and tumors. Second, regardless of the training, test or external test sets, the sample size was small. Thus, these results need to be validated with a larger cohort to determine the value of our model in clinical practice. Third, because the study was retrospective, all patients underwent surgical treatment. However, there are many women who have a BI-RADS categorization with certain malignant potential who choose observation instead of surgical treatment. This factor may be one of the reasons why our study did not achieve a better result.

## Conclusion

We demonstrated that our DLM can recognize breast tumors and predict molecular subtypes with high accuracy based solely on ultrasound images, which may make DLM an effective alternative to clinical biopsy. It is necessary to cooperate with other institutions to expand the dataset to better confirm our model and make it an important decision-making tool with great potential in clinical application.

## Data Availability Statement

The original contributions presented in the study are included in the article/Supplementary Material, further inquiries can be directed to the corresponding author/s.

## Ethics Statement

This study was approved by the Institutional Review Board of Harbin Medical University Cancer Hospital. Because of its retrospective nature, the written informed consent of patients was exempted.

## Author Contributions

XZ, YaZ, HM, and DP conceived and designed the study. WC, HJ, SL, and JH collected the clinical and imaging data. HL, JL, and YL performed image preprocessing. XZ, CW, YuZ, DL, YaZ, HM, and DP performed the data interpretation and the statistical analysis. All authors approved the final manuscript.

## Conflict of Interest

The authors declare that the research was conducted in the absence of any commercial or financial relationships that could be construed as a potential conflict of interest.
