# Supplementary material for: Evaluating the Accuracy of Breast Cancer and Molecular Subtype Diagnosis by Ultrasound Image Deep Learning Model
Source: Front Oncol. 2021 Mar 5;11:623506. doi: 10.3389/fonc.2021.623506 (PMC7973262; doi:10.3389/fonc.2021.623506)
Supplement: Supplementary file 1 [file Table_1.DOCX]

**Supplementary Methods**

**Exclusion criteria for the data sets**

First, we selected the patients. We excluded patients whose pathological results were neither benign nor malignant (such as mammary gland hyperplasia and fat necrosis). Patients with BI-RADS 1 or 2 and abnormal mammography results were also excluded. Patients who were diagnosed with Paget's disease but had no masses in the breasts were also excluded. Second, we filter the images. We deleted the ultrasound images of lymph nodes, color Doppler ultrasound images, normal breast images (images with no masses) and ultrasound images with tumors but without surgical resection.

**Image pre-processing process**

In the first stage, we cropped the effective region of the images and selected the tumor region of the ultrasound images. Then, the minimum region of interest was selected when all the information of the tumor was included as much as possible to reduce the difficulty of algorithm recognition. This operation was completed by experienced radiologists, and three radiologists independently evaluated the image by determining a BI-RADS categorization. After combining three results, a definite BI-RADS categorization was obtained for each tumor. In the second stage, we cropped the selected effective image areas. Because of the existence of medical marks, to restore the original image of the ultrasound images and reduce the interference of the marks, we used the image repair algorithm proposed by Telea and others to remove the interference marks of the area of interest and used the similar texture of the surrounding area to restore the gap (1). As shown in Supplementary Figure 2, image pre-processing was performed from the original image to the repaired breast ultrasound images of interest.

**The training parameters**

The Supplementary Table 2 shows our training parameters. Each network is divided into 2 parts: Transfer and Fine-tune. In the Transfer part, we select the iterative and fast Adam optimizer algorithm (2). The learning rate was set to 0.01, and the epoch of training was set to 20. In the Fine-tune section, we selected SGD algorithm, with the learning rate set at 0.001 and the number of training times set at 200.On CNN-3, the other parameters were the same as CNN-2, and the fine-tune epoch was set as 100.

**Sample size calculation**

A general sample size formula for comparison of proportion with fixed value can be applied for evaluation of single diagnostic test. With $(1-\alpha)$ confidence level and$(1-\beta)$ power for detection an effect of $P_{1}-P_{0}$ using normal approximation as a general rule, Z-score under the null and alternative hypothesis can defined and thus the required sample size for cases is driven as follows:

$$n=\frac{\left[ Z_{\frac{\alpha}{2}}\sqrt{P_{0}\left( 1-P_{0} \right)}+Z_{\beta}\sqrt{P_{1}\left( 1-P_{1} \right)} \right]^{2}}{\left( P_{1}-P_{0} \right)^{2}}$$

where $Z_{\frac{\alpha}{2}}$ and $Z_{\beta}$denote the upper $\frac{\alpha}{2}$ and $\beta$ percentiles of standard normal distribution and $\alpha$, $\beta$are the probability of type I and type II errors respectively. For $\alpha=0.05$and$\beta=0.20$, they are inserted by$Z_{\frac{\alpha}{2}}=1.96$ and $Z_{\beta}=0.84$ respectively.

The pre-determined value of sensitivity or specificity was estimated as 80%. The sample size one would need to have 95% confidence and 80% power to detect a difference of 10% from pre-determined value of $Se/Spe=0.80$, can be calculated as follows:

$n=\frac{(1.96\times\sqrt{0.80\times0.20}+0.84\times\sqrt{0.70\times0.30})^{2}}{(0.10)^{2}}=153$(3)

Hence, sample size of our study (a total of 17226 images from 2542 patients) have enough power to support the conclusion.

**Supplementary Figure Legends**

**Supplementary Figure 1:** Flow chart of breast ultrasound image analysis based on the migration and fine-tuning of the Xception network.

**Supplementary Figure 2:** The process of image pre-processing, from the original image to the repaired breast tumor ultrasound image of interest.

**Supplementary Table 1:** Confusion matrix of the molecular subtypes by triple classification in the test set.

| **Pathology**  **DLM** | **Her2 (+)** | **HR (+)** | **Triple-negative** | **Total** |
| --- | --- | --- | --- | --- |
| **Her2 (+)** | 27 | 14 | 4 | 45 |
| **HR (+)** | 22 | 126 | 18 | 166 |
| **Triple-negative** | 5 | 8 | 25 | 38 |
| **Correct** | 27 | 126 | 25 | 178 |
| **Total** | 54 | 148 | 47 | 249 |
| **Accuracy rate (%)** | 50.00 | 85.14 | 53.19 | 71.49 |

Note: HER2 (+) =HR (+) HER2 (+) or HR (-) HER2 (+); HR (+) = HR (+) HER2 (-); triple-negative = HR (-) HER2 (-).

**Supplementary Table 2:** **The training parameters**

| Model/Parameter | Optimizer | Learning rate | Batch size | Epoch |
| --- | --- | --- | --- | --- |
| CNN-2 | Adam,SGD | 0.01,0.001 | 32 | 20,200 |
| CNN-3 | Adam,SGD | 0.01,0.001 | 32 | 20,100 |

**Supplementary Reference**

1. Telea, Alexandru. An Image Inpainting Technique Based on the Fast Marching Method. Journal of Graphics Tools. (2004) 9:23-34. doi: 10.1080/10867651.2004.10487596

2. Kingma DP, Ba J. Adam: A Method for Stochastic Optimization. *Computer Ence*. (2014)

3. Hajian-Tilaki K. Sample size estimation in diagnostic test studies of biomedical informatics. *J Biomed Inform*. (2014) 48:193-204. doi: 10.1016/j.jbi.2014.02.013
