# Supplementary figures and images for: Evaluating the Accuracy of Breast Cancer and Molecular Subtype Diagnosis by Ultrasound Image Deep Learning Model
Source: Front Oncol. 2021 Mar 5;11:623506. doi: 10.3389/fonc.2021.623506 (PMC7973262; doi:10.3389/fonc.2021.623506)

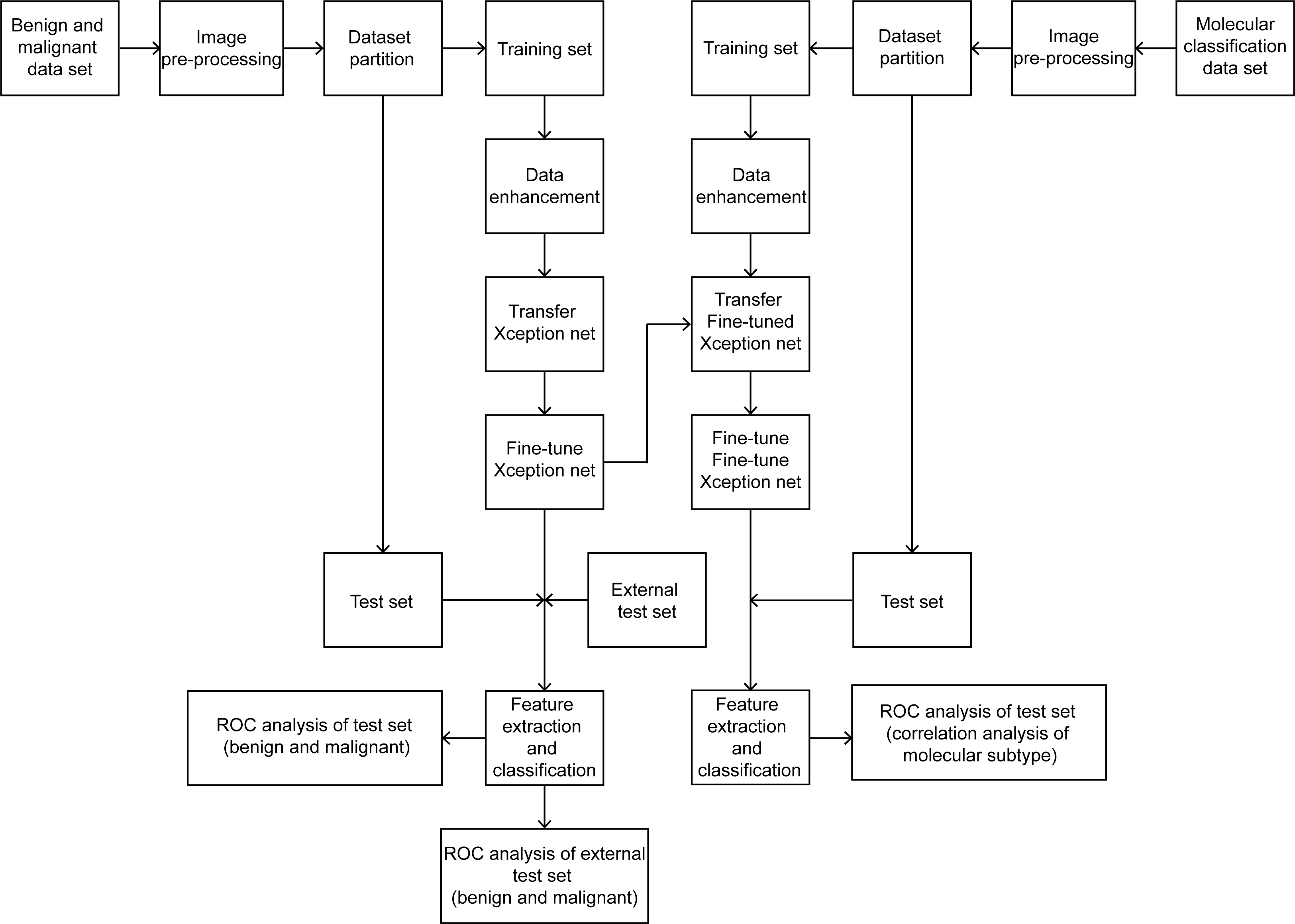

Supplement: Supplementary file 2 [file Image_1.TIF]

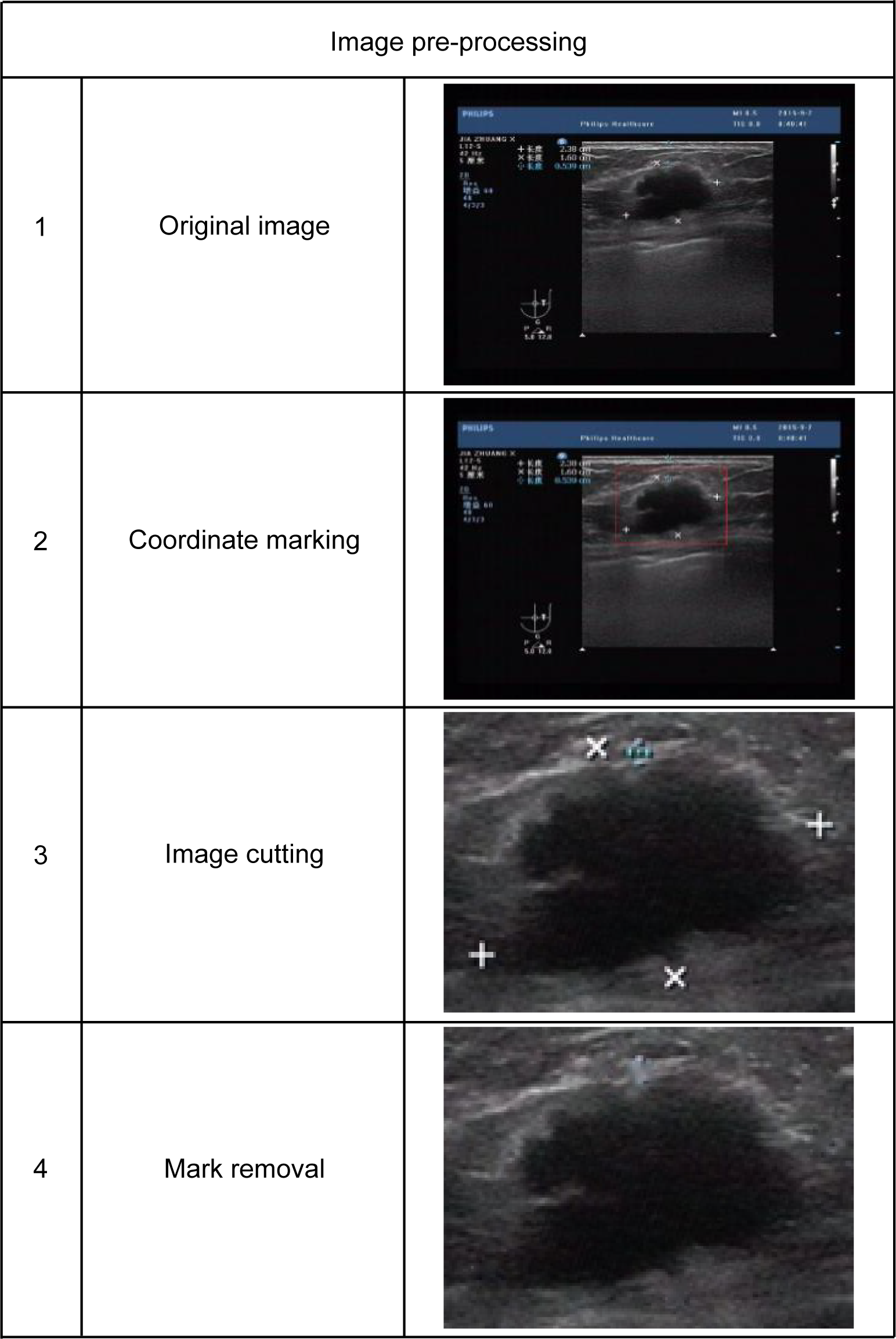

Supplement: Supplementary file 3 [file Image_2.TIF]
